# Genome-wide significance for a modifier of age at neurological onset in Huntington's Disease at 6q23-24: the HD MAPS study

**DOI:** 10.1186/1471-2350-7-71

**Published:** 2006-08-17

**Authors:** Jian-Liang Li, Michael R Hayden, Simon C Warby, Alexandra Durr, Patrick J Morrison, Martha Nance, Christopher A Ross, Russell L Margolis, Adam Rosenblatt, Ferdinando Squitieri, Luigi Frati, Estrella Gómez-Tortosa, Carmen Ayuso García, Oksana Suchowersky, Mary Lou Klimek, Ronald JA Trent, Elizabeth McCusker, Andrea Novelletto, Marina Frontali, Jane S Paulsen, Randi Jones, Tetsuo Ashizawa, Alice Lazzarini, Vanessa C Wheeler, Ranjana Prakash, Gang Xu, Luc Djoussé, Jayalakshmi Srinidhi Mysore, Tammy Gillis, Michael Hakky, L Adrienne Cupples, Marie H Saint-Hilaire, Jang-Ho J Cha, Steven M Hersch, John B Penney, Madaline B Harrison, Susan L Perlman, Andrea Zanko, Ruth K Abramson, Anthony J Lechich, Ayana Duckett, Karen Marder, P Michael Conneally, James F Gusella, Marcy E MacDonald, Richard H Myers

**Affiliations:** 1 Department of Neurology, Boston University School of Medicine, Boston, MA, USA; 2 Bioinformatics Program, Boston University, Boston, MA, USA; 3 Department of Biological Technologies, Wyeth Research, Cambridge, MA, USA; 4 Centre for Molecular Medicine & Therapeutics and Department of Medical Genetics, University of British Columbia, Vancouver, British Columbia, Canada; 5 INSERM U679, Hôpital de la Salpêtrière, Paris, France; 6 Department of Medical Genetics, Belfast City Hospital, Belfast, UK; 7 School of Biomedical Science, University of Ulster, Coleraine, UK; 8 Department of Neurology, Hennepin County Medical Center, Minneapolis, Minnesota, USA; 9 Departments of Psychiatry and Neurology, John Hopkins University, Baltimore, Maryland, USA; 10 Program in Cellular and Molecular Medicine, John Hopkins University, Baltimore, Maryland, USA; 11 Department of Neuroscience, John Hopkins University, Baltimore, Maryland, USA; 12 Neurogenetics Unit, IRCCS Neuromed, Pozzilli, Italy; 13 Dept of Experimental Medicine and Pathology, University "La Sapienza" of Rome, Rome, Italy; 14 Servicio de Neurología y Genética, Fundación Jiménez Díaz, Madrid, Spain; 15 Departments of Clinical Neurosciences and Medical Genetics, University of Calgary, Calgary, Alberta, Canada; 16 Department of Medicine, University of Sydney, Sydney, Australia; 17 Neurology Department, Westmead Hospital, Sydney, Australia; 18 Department of Biology, University "Tor Vergata", 00133 Rome, Italy; 19 Institute of Neurobiology and Molecular Medicine, CNR, Rome, Italy; 20 Department of Psychiatry, University of Iowa, Iowa City, Iowa, USA; 21 Neurology Department, Emory University, Atlanta, Georgia, USA; 22 Department of Neurology, University of Texas Medical Branch, Galveston, TX, USA; 23 Department of Neurology, Robert Wood Johnson school of Medicine and Dentistry of New Jersey, USA; 24 Novartis Pharmaceuticals, New Brunswick, NJ, USA; 25 Molecular Neurogenetics Unit, Center for Human Genetic Research, Massachusetts General Hospital, Boston, MA, USA; 26 Section of Preventive Medicine and Epidemiology, Evans Department of Medicine, Boston University School of Medicine, Boston, MA, USA; 27 Department of Biostatistics, School of Public Health, Boston University, Boston MA, USA; 28 Department of Neurology, Massachusetts General Hospital, Boston, MA, USA; 29 Health Sciences Center, University of Virginia, Charlottesville, Virginia, USA; 30 Department of Neurology, University of California at Los Angeles, California, USA; 31 Division of Medical Genetics, UCSF, San Francisco, California, USA; 32 WMS Hall Psychiatric Institute, Columbia, South Carolina, USA; 33 Department of Neurology, Columbia College of Physicians, New York, NY, USA; 34 Department of Genetics, Indiana University School of Medicine, Indianapolis, IN, USA; 35 Department of Genetics, Harvard Medical School, Boston, MA, USA

## Abstract

**Background:**

Age at onset of Huntington's disease (HD) is correlated with the size of the abnormal CAG repeat expansion in the *HD *gene; however, several studies have indicated that other genetic factors also contribute to the variability in HD age at onset. To identify modifier genes, we recently reported a whole-genome scan in a sample of 629 affected sibling pairs from 295 pedigrees, in which six genomic regions provided suggestive evidence for quantitative trait loci (QTL), modifying age at onset in HD.

**Methods:**

In order to test the replication of this finding, eighteen microsatellite markers, three from each of the six genomic regions, were genotyped in 102 newly recruited sibling pairs from 69 pedigrees, and data were analyzed, using a multipoint linkage variance component method, in the follow-up sample and the combined sample of 352 pedigrees with 753 sibling pairs.

**Results:**

Suggestive evidence for linkage at 6q23-24 in the follow-up sample (LOD = 1.87, *p *= 0.002) increased to genome-wide significance for linkage in the combined sample (LOD = 4.05, *p *= 0.00001), while suggestive evidence for linkage was observed at 18q22, in both the follow-up sample (LOD = 0.79, *p *= 0.03) and the combined sample (LOD = 1.78, *p *= 0.002). Epistatic analysis indicated that there is no interaction between 6q23-24 and other loci.

**Conclusion:**

In this replication study, linkage for modifier of age at onset in HD was confirmed at 6q23-24. Evidence for linkage was also found at 18q22. The demonstration of statistically significant linkage to a potential modifier locus opens the path to location cloning of a gene capable of altering HD pathogenesis, which could provide a validated target for therapeutic development in the human patient.

## Background

Huntington's disease (HD [MIM 143100]) is a progressive neurodegenerative disorder with an age at neurological onset commonly in midlife. The major clinical features of HD include involuntary choreiform movements, psychiatric symptoms, and cognitive dysfunction [1-3]. The genetic mutation associated with HD is located in 4p16.3 and is characterized by expansion of a CAG repeat in the first exon of the gene encoding the huntingtin protein [4]. Many studies have examined the relationship of the CAG repeat to neurological onset in HD, and found that its length accounts for about 70% of the variation in age at onset [5,6]. Our recent studies [7] and those of others [5,6] suggest that the remaining variation in HD age at onset is strongly heritable and about 56% [8] of the variance remaining in age at onset is attributable to genes other than the *HD *gene, supporting the existence of genes capable of modifying HD pathogenesis. Although Wexler et al. [5] suggest that 60% of the variance may be attributable to environmental factors, remarkable similarity for onset age in monozygotic twins [3] support primarily genetic modifiers for this trait.

Identification of genetic modifiers in HD could be of enormous importance for defining the mechanisms that may be capable of delaying the onset of the disorder. We recently reported a whole-genome scan for modifiers of age at onset for HD in 295 pedigrees containing 629 sibling pairs, with six regions, 2q33, 4p16, 5q31-32, 6p22, 6q23-24, and 18q22 exhibiting LOD scores > 1.5 [8]. In the present study, we sought to confirm our original whole-genome scan findings by conducting a follow-up study of the peak regions observed in the original scan using a newly recruited expanded follow-up sample.

## Methods

### Subjects

Three sample sets, newly recruited (Follow-up Sample), original (Original Sample) and combined (Combined Sample) were used in this study. Prior to the data cleaning, the Follow-up Sample consists of 149 newly recruited HD patients. Fifteen of the newly recruited siblings were members of 12 pedigrees used in the Original Study [8]. For these individuals a single sibling was randomly selected from the Original Study pedigree to create a sibling pair for the Follow-up study. The remaining 134 new patient samples were recruited from 61 new pedigrees. Only 57 of the newly recruited pedigrees, with 126 siblings were kept after removing three apparently identical twin pairs and one pair lacking onset information. Thus, the final Follow-up Sample contained 69 pedigrees (12 original and 57 newly recruited) with 141 (15 + 126) newly recruited subjects and 102 sibling pairs (Table 1).

**Table 1:** The Study Subjects.

	**Original Sample**	**Follow-up Sample**	**Combined Sample**
Pedigrees	295	69^*a*^	352
Sibling-pairs	629	102	753^*b*^
Patients	695	141	836
Mean Onset ± SD	39.3 ± 12.1	39.2 ± 11.8	39.3 ± 12.0
Mean HD repeat ± SD	46.4 ± 5.9	45.5 ± 5.5	46.2 ± 5.7

Note (details provided in Subjects section of Materials and Methods):

^*a *^57 pedigrees are newly recruited, and the remaining 12 are from the Original Sample with newly recruited siblings.

^*b *^The number of pairs in the Combined Sample exceeds that for the sum of the Original Sample and Follow-up Sample because additional sibling pairs (21 new sibling pairs and one half sib) were created when new siblings were added to existing pedigrees.

The Original Sample, used in the genome scan paper [8], consisted of 295 pedigrees with 629 sibling pairs. The Original Sample contained 20 unaffected parents and 9 unaffected siblings to increase precision in the estimation of identity by descend [8]. The Combined Sample, consisting of both the Original Sample and the Follow-up Sample, had 352 pedigrees with 836 HD subjects. Twenty-one new sibling pairs and one half sib-pair were created when new siblings were added to existing pedigrees for a total of 753 sibling pairs in the Combined Sample (Table 1).

### Age at onset

Age at onset, defined as the onset of motor impairment, was reported for all affected participants [9,10]. Cases with 36 or more repeats, were designated *HD *mutation carriers in accordance with published associations with disease expression [11]. The quantitative trait utilized in linkage analysis was adjusted for the effects of the CAG repeat expansion using two different regression models. Both models used the logarithmically transformed age at onset as the dependent variable. The first model (Model One) adjusted only for the size of the expanded CAG repeat [log(onset) = α + β(HD)CAG]. The second model (Model Two), that we had used in our original genome scan [8] and previously described [7], adjusted for the HD repeat, the normal repeat and their interaction [log(onset) = α + β_1_(HD)CAG + β_2_(Normal)CAG + β_3_(HD)CAG × (Normal)CAG]. Random effect models (*Proc MIXED *in SAS) were used in these models to account for familial clustering. Each model was used to determine the expected age at onset for a given expanded CAG repeat, and the residual was computed as the difference between the observed and expected age at onset. Residuals were standardized to a mean of zero and a standard deviation of 1. Both models show similar modest negative skewness. The skewness of the residual generated by Model One is -0.42 and the kurtosis is 1.82. Corresponding values for Model Two were -0.49 and 1.11, respectively.

Residual onset ages for all analyses were computed using the 836 combined sample plus 234 locally studied HD patients recruited from the New England HD Research Center and 303 brain specimens from the McLean Brain Tissue Resource Center, for a total sample of 1373. While the local and brain samples are not included in this linkage analysis, they provide an additional randomly ascertained samples to more accurately model the relationship between age at onset and CAG repeat sizes. Finally, because our future studies will involve SNP association studies in all of three samples (HD MAPS families, the locally collected DNAs, and the brain specimens), we sought to define the repeat adjusted age at onset uniformly across all three samples.

The heritability analyses were conducted using maximum likelihood procedures as implemented in the SOLAR program [12].

### CAG repeat size determination

*HD *CAG repeat sizes were determined by polymerase chain reaction using an assay that does not include the adjacent proline (CCG) repeat. Cases with 36 or more repeats were designated *HD *mutation carriers.

### Genotyping

All the newly recruited HD samples were genotyped by three microsatellite markers at each of the six regions with maximum multipoint LOD scores greater than or equal to 1.5 in the original genome scan [8]. The markers at each locus comprised the peak marker and the two markers from the original scan that flank it (see Table 2). Prior to the analysis, the sib_kin program in the ASPEX package [13] was used to verify sibling relationships. Mendelian inconsistencies were then identified using INFER, in the PEDSYS package [14] and MERLIN [15]. Genotypes for the entire nuclear family were deleted for the particular marker when an inconsistency was detected. The genotyping data set was 93% complete, with 7% genotyping failure or error rate.

**Table 2:** Eighteen microsatellite markers genotyped, three at each of the six loci, for Follow-up study. Marker 2 is the peak marker in original study at each locus.

**Chromosome**	**Marker 1**	**Position (cM)**	**Marker 2**	**Position (cM)**	**Marker 3**	**Position (cM)**
2q33	*D2S1391*	186	*D2S1384*	200	*D2S2944*	210
4p16	*D4S3360*	0	*D4S2366*	13	*D4S403*	26
5q31-32	*D5S816*	139	*D5S1480*	147	*D5S820*	160
6p22.3	*D6S1006*	27	*D6S1959*	34	*D6S2439*	42
6q23-24	*D6S1009*	138	*GATA184A08*	146	*D6S2436*	155
18q22	*D18S851*	75	*D18S858*	80	*D18S1357*	89

### Linkage analysis

Variance component linkage analysis to repeat adjusted age at onset was performed using MERLIN [15]. We performed multipoint linkage analysis in the Original 295 pedigrees, the Follow-up 69 pedigrees, and the Combined 352 pedigrees. Adjusted age at onset was available for all HD affected participants. Age at onset was coded as "missing" for all unaffected individuals.

The oligogenic linkage analysis and epistatic interaction analysis were performed by the SOLAR program [12]. MERLIN was used to generate the IBD estimates and these were converted into SOLAR format for analyses. The viability of epistatic model was tested. The interaction term was constrained to non-interaction, and then the difference between interaction and non-interaction models was tested by chi-square.

## Results

Three sample sets, newly recruited (Follow-up Sample), original (Original Sample) and combined (Combined Sample) were used in this study. The Original, Follow-up and Combined samples are described in Table 1. The mean age at onset is similar for the Follow-up (range 17 to 70 y) and Original samples (range 9 to 82 y). All three samples exhibited strong heritability estimates. The heritability estimates (*h*^2 ^± SE) for expanded repeat adjusted age at onset (Model One) are 0.72 ± 0.09 for the Original Sample, 0.74 ± 0.20 for the Follow-up Sample and 0.74 ± 0.08 for the Combined Sample.

**Figure 1:**
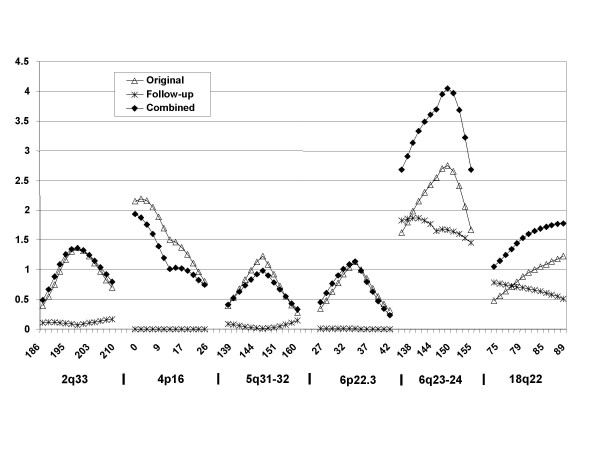
Multipoint linkage results generated by MERLIN across six chromosome regions in followup study. The x-axis indicates genetic distance and the y-axis indicates LOD score. These data show the confirmed evidence for linkage to 6q23-24 using Model One age at onset adjustment.

Multipoint linkage analyses were completed using both Model One, adjusting for expanded HD repeats only, and Model Two, adjusting for expanded HD repeats, normal repeats and their interactions. Multipoint LOD scores obtained in these three sample sets for the six chromosome regions (2q33, 4p16, 5q31-32, 6p22, 6q23-24, and 18q22) are presented in Table 3 and Figure 1. For the Follow-up Sample, the highest LOD score was observed at the 6q23-24 region (LOD = 1.87, *p *= 0.002, Model One; or LOD = 2.27, *p *= 0.0006, Model Two). One additional region provided modest confirmation for linkage, 18q22 (LOD = 0.79, *p *= 0.03, Model One; or LOD = 0.79, *p *= 0.02, Model Two). However, no evidence for linkage was seen at 2q33 (LOD = 0.17, *p *= 0.2, Model One; LOD = 0.21, *p *= 0.2, Model Two), 4p16 (LOD = 0.0, *p *= 0.5, Model One and Model Two), 5q31-32 (LOD = 0.15, *p *= 0.2, Model One; LOD = 0.12, *p *= 0.2, Model Two), and 6p22 (LOD = 0.01, *p *= 0.4, Model One and Model Two).

**Table 3:** Multipoint LOD score and chromosomal location in the Original, Follow-up and Combined samples are shown.

**A **Model One LOD scores.									
Chromosome Location	Original Sample			Follow-up Sample			Combined Sample		
			
	Distance * (cM)	LOD	*p*-value	Distance * (cM)	LOD	*p*-value	Distance * (cM)	LOD	*p*-value
		
2q33	200	1.37	0.006	210	0.17	0.2	200	1.37	0.006
4p16	2	2.19	0.0007	0	0	0.5	0	1.94	0.0014
5q31-32	147	1.23	0.009	160	0.15	0.2	142	0.98	0.02
6p22.3	34	1.13	0.011	34	0.01	0.4	34	1.14	0.011
6q23-24	149	2.75	0.0002	142	1.87	0.002	149	4.05	0.00001
18q22	89	1.23	0.009	75	0.79	0.03	89	1.78	0.002

**B **Model Two LOD scores.									

2q33	200	1.56	0.004	210	0.21	0.2	200	1.62	0.003
4p16	2	2.15	0.0008	0	0	0.5	0	1.9	0.002
5q31-32	147	1.27	0.008	160	0.12	0.2	147	0.98	0.02
6p22.3	34	0.88	0.02	34	0.01	0.4	34	0.95	0.02
6q23-24	148	3.5	0.00003	142	2.27	0.0006	149	4.94	<10^-6^
18q22	89	0.96	0.02	75	0.79	0.02	89	1.55	0.004

A. Model One was used to adjust age at onset and age at onset was adjusted by HD repeats only; B. Model Two was used to adjust age at onset, and age at onset was adjusted by HD repeat, normal repeat and their interaction.

* The genetic distances are as indicated by the Marshfield linkage map [26]

Model One analysis in the Combined Sample yielded significant linkage at 6q23-24 (LOD = 4.05, *p *= 0.00001) and suggestive linkage at 4p16 (LOD = 1.94, *p *= 0.0014) and 18q22 (LOD = 1.78, *p *= 0.002). The 2q33 (LOD = 1.37, *p *= 0.006) and 6p22.3 (LOD = 1.14, *p *= 0.011) regions achieved a LOD score greater than 1.0. However, the LOD score at 5q31-32 dropped to 0.98 (*p *= 0.02). The Model Two analysis, adjusting for the expanded repeat, normal repeats and their interaction, generated higher LOD scores than Model One at 6q23-24 in all the three tested sample sets: the Original Sample (LOD = 3.5, *p *= 0.00003), Follow-up Samples (LOD = 2.27, *p *= 0.0006) and Combined Sample (LOD = 4.94, *p *< 10^-6^). All of the other analyses using Model Two produced LOD scores very similar to those of Model One. Epistatic analysis indicated that there is no interaction between 6q23-24 and five other loci.

## Discussion

The purpose of this study was to replicate our original linkage findings for repeat adjusted age at onset in HD [8] in an expanded sample. A *p *value of 0.01 is needed to confirm evidence for linkage [16]. In this Follow-up study, strong evidence for linkage was observed at 6q23-24 (*p *= 0.002) and suggestive support for linkage was observed at 18q22 (*p *= 0.03). No evidence for linkage was observed in the Follow-up Sample at 2q33 (*p *= 0.2), 4p16 (*p *= 0.5), 5q31-32 (*p *= 0.2) and 6p22.3 (*p *= 0.4). The lack of confirmation for the latter four loci may indicate that these regions do not contain genes that modify the age at neurologic onset for HD or that this relatively small Follow-up study did not adequately sample families which carry modifier genes from these regions.

In the Combined Sample, the Model One analysis, adjusting only for the size of the expanded repeat, yielded significant linkage at 6q23-24 (LOD = 4.05, *p *= 0.00001). The Model Two analysis, adjusting for the expanded repeat, normal repeats and their interaction, generated higher LOD score than Model One at 6q23-24. The LOD scores of the Original Sample (LOD = 3.5, *p *= 0.00003), Follow-up Samples (LOD = 2.27, *p *= 0.0006) and Combined Sample (LOD = 4.94, *p *< 10^-6^) are highly significant using this second model. All of the other analyses using Model Two produced LOD scores very similar to those of Model One. We emphasize results from Model One because beta coefficients from Model Two show greater variability compared with those computed in the original scan [8]. Changes in the composition of the sample have modified the relationship of the repeat sizes to onset age from that seen in the original sample alone [8]. Although all three terms in the model (HD repeat, normal repeat and the interaction of these) are significant predictors of age at onset, the sign of the beta coefficients were opposite to those seen in the original scan [8] for the normal repeat and interaction terms. Consequently, the Model Two adjustment may be susceptible to as yet unidentified sample stratification effects or over-specification of the model.

The results of the Combined Sample are generally similar to those of the original genome scan (see Table 3). Significant evidence for linkage was observed at 6q23-24 in the Combined Sample (LOD = 4.05, *p *= 0.00001). We reported a LOD score of 2.28 at 6q23-24 in our original genome scan [8], while the same sample generates a LOD score of 3.5 (*p *= 0.00003) in the current study using the same model (Model Two). The difference is due to a modification of the method used to define the repeat adjusted age at onset. In the original genome scan only the 754 individuals were used to model the relationship of repeat size age at onset. In the present study, we used a sample of 1373 individuals, derived from the 836 in the combined sample, plus 234 locally studied HD affected persons and a sample of 303 brain specimens. While the local and brain samples do not represent sib-pairs that could be included in this linkage analysis, they provide additional randomly ascertained samples to more accurately model the relationship between age at onset and CAG repeat sizes. They also provide an increased sample size for subsequent fine-mapping association studies to assess candidate modifiers. The increased sample size provided a more accurate assessment of the relationship between repeat size and age at onset, yielding a residual that more accurately adjusts for the effect of repeat size on age at onset in HD.

In addition to the 6q23-24 peak, the Follow-up study supports evidence for linkage at 18q22 (LOD = 0.79, *p *= 0.03, Model One; LOD = 0.79, *p *= 0.02, Model Two). The Combined Sample provides suggestive evidence for linkage at this locus (LOD = 1.78, *p *= 0.002, Model One; LOD = 1.55, *p *= 0.004, Model Two). Suggestive linkage is still observed at 4p16 (LOD = 1.94, *p *= 0.0014, Model One; LOD = 1.9, *p *= 0.002, Model Two) in the Combined Sample, although, this locus was not confirmed in the Follow-up study and the LOD score is lower than that of the original scan (LOD = 2.19, *p *= 0.0007, Model One; LOD = 2.15, *p *= 0.0008, Model Two). The decreased LOD score at 4p16 may be a consequence of genetic heterogeneity, possibly reflecting the diverse ethnic background of the sample [17].

The epistatic analysis indicated that there is no interaction between 6q23-24 with five other loci. Therefore, we assumed that the potential modifiers are not members in a common pathway. The 1-LOD unit support interval (133 – 153 Mb) at 6q23-24 contains 128 known and predicted genes (Ensembl v27) [18]. Two genes of particular interest are serum and glucocorticoid regulated kinase (*SGK*, 135 Mb) and metabotropic glutamate receptor 1 (*GRM1*, 146 Mb). A recent study reported that *SGK *levels are increased in brains of HD patients; *SGK *phosphorylates huntingtin at serine 421, protecting striatal neurons against toxicity caused by a polyQ-huntingtin amino-terminal fragment [19]. *SGK *is a plausible candidate gene. *GRM1 *is located within 2 Mb of the peak marker (GATA184A08, 148 Mb) and is highly expressed in the cerebellum [20]. One prominent action of *GRM1 *is to protect neurons from apoptotic death [21]. In addition, several studies reported that *GRIK2 *is associated with early onset [22,23]. *GRIK2 *(6q16.3, 102 Mb) is about 30 Mb proximal to the 1-LOD interval on the confirmed 6q23-24 (133 – 153 Mb). However, the variance in onset age explained by *GRIK2 *is small and one would not expect that it would be detected by linkage. The 7-Mb 1-LOD unit support interval (50 – 57 Mb) at 18q22 contains 36 known genes, according to the Ensembl database (v27) [18]. Interesting candidate genes in this interval include *NEDD4L *(18q21, 54 Mb), which encodes a neural precursor cell expressed, developmentally down-regulated 4-like gene. *NEDD4L *is an ubiquitin ligase and contains WW domains. Yeast two-hybrid studies found that huntingtin binds to a group of genes with WW domains [24]. One of the interesting features of *NEDD4L *is that it may mediate degradation of the product of *SGK*, the above mentioned candidate gene located at 6q23 [25].

## Conclusion

In conclusion, this replication study confirms evidence for linkage in the 6q23-24 region observed in our original genome scan. Although the other regions, particularly 18q22, may also contain genes that modify age at onset in HD, the 6q23-24 shows evidence for harboring one or more genetic modifiers that exceeds the level required for genome-wide statistical significance (LOD >3.6) [16]. In our approach, a genetic modifier of HD is a gene that is inherently capable of modifying the course of disease pathogenesis, thereby altering the observed age at onset. Consequently, identifying such genetic modifiers is a potential route to validated targets for therapeutic development aimed at delaying or preventing neurological onset in HD. We present evidence that the 6q23-24 region contains such a genetic modifier, which opens the way for its identification and eventual exploitation for treatment of this devastating disorder.

## Abbreviations

HD: Huntington's disease

HD MAPS: Huntington's Disease Modifiers of Age at Onset in Pairs of Siblings

QTL: Quantitative Trait Loci

LOD: logarithm of the odds

## Competing interests

The author(s) declare that they have no competing interests.

## Authors' contributions

JL participated in the design of the study, performed the statistical analysis and drafted the manuscript. RP was responsible for the coordinating the study groups and sample collection. MRH, SCW, AD, PJM, MN, CAR, RLM, AR, FS, LF, EG, CAG, OS, MLK, RJT, EM, AN, MF, JSP, RJ, TA, AL, MHS, JJC, SMH, JBP, MBH, SLP, AZ, RKA, AJL, AD, KM, and PMC had ascertained the clinical status of the patients and provided the patient samples. JSM, TG, and MH were responsible for the genotyping. GX and LD participated in the data analysis. LAC, VCW, JFG and MEM participated in the study design, data generation, data analysis and manuscript preparation. RHM was responsible for study conception, design, and oversight and finalized the data analysis as well as manuscript preparation. All authors read and approved the final manuscript.

## Pre-publication history

The pre-publication history for this paper can be accessed here:



## References

[B1] Bates G, Harper P, Jones L (2002). Huntington's Disease.

[B2] Myers RH, Marans K, Macdonald ME, Warren St, Wells RT (1998). Huntington's disease. Genetic Instabilities and Hereditary Neurological Diseases.

[B3] Hayden MR (1981). Huntington's Chorea.

[B4] The Huntington's Disease Collaboratide Research Group (1993). A novel gene containing a trinucleotide repeat that is expanded and unstable on Huntington's disease chromosomes.. Cell.

[B5] Wexler NS, Lorimer J, Porter J, Gomez F, Moskowitz C, Shackell E, Marder K, Penchaszadeh G, Roberts SA, Gayan J, Brocklebank D, Cherny SS, Cardon LR, Gray J, Dlouhy SR, Wiktorski S, Hodes ME, Conneally PM, Penney JB, Gusella J, Cha JH, Irizarry M, Rosas D, Hersch S, Hollingsworth Z, MacDonald M, Young AB, Andresen JM, Housman DE, De Young MM, Bonilla E, Stillings T, Negrette A, Snodgrass SR, Martinez-Jaurrieta MD, Ramos-Arroyo MA, Bickham J, Ramos JS, Marshall F, Shoulson I, Rey GJ, Feigin A, Arnheim N, Acevedo-Cruz A, Acosta L, Alvir J, Fischbeck K, Thompson LM, Young A, Dure L, O'Brien CJ, Paulsen J, Brickman A, Krch D, Peery S, Hogarth P, Higgins DSJ, Landwehrmeyer B (2004). Venezuelan kindreds reveal that genetic and environmental factors modulate Huntington's disease age of onset. Proc Natl Acad Sci U S A.

[B6] Rosenblatt A, Brinkman RR, Liang KY, Almqvist EW, Margolis RL, Huang CY, Sherr M, Franz ML, Abbott MH, Hayden MR, Ross CA (2001). Familial influence on age of onset among siblings with Huntington disease. Am J Med Genet.

[B7] Djoussé L, Knowlton B, Hayden M, Almqvist EW, Brinkman R, Ross C, Margolis R, Rosenblatt A, Durr A, Dode C, Morrison PJ, Novelletto A, Frontali M, Trent RJ, McCusker E, Gomez-Tortosa E, Mayo D, Jones R, Zanko A, Nance M, Abramson R, Suchowersky O, Paulsen J, Harrison M, Yang Q, Cupples LA, Gusella JF, MacDonald ME, Myers RH (2003). Interaction of normal and expanded CAG repeat sizes influences age at onset of Huntington disease. Am J Med Genet A.

[B8] Li JL, Hayden MR, Almqvist EW, Brinkman RR, Durr A, Dode C, Morrison PJ, Suchowersky O, Ross CA, Margolis RL, Rosenblatt A, Gomez-Tortosa E, Cabrero DM, Novelletto A, Frontali M, Nance M, Trent RJ, McCusker E, Jones R, Paulsen JS, Harrison M, Zanko A, Abramson RK, Russ AL, Knowlton B, Djousse L, Mysore JS, Tariot S, Gusella MF, Wheeler VC, Atwood LD, Cupples LA, Saint-Hilaire M, Cha JH, Hersch SM, Koroshetz WJ, Gusella JF, MacDonald ME, Myers RH (2003). A genome scan for modifiers of age at onset in Huntington disease: The HD MAPS study. Am J Hum Genet.

[B9] Conneally PM (1984). Huntington disease: genetics and epidemiology. Am J Hum Genet.

[B10] Farrer LA, Conneally PM (1987). Predictability of phenotype in Huntington's disease. Arch Neurol.

[B11] Duyao M, Ambrose C, Myers R, Novelletto A, Persichetti F, Frontali M, Folstein S, Ross C, Franz M, Abbott M (1993). Trinucleotide repeat length instability and age of onset in Huntington's disease. Nat Genet.

[B12] Almasy L, Blangero J (1998). Multipoint quantitative-trait linkage analysis in general pedigrees. Am J Hum Genet.

[B13] Hinds D, Risch N (1999). The ASPEX package: affected sib-pair exclusion mapping. http://aspex.sourceforge.net.

[B14] PEDSYS PEDSYS package. http://www.sfbr.org/software/pedsys/pedsys.html.

[B15] Abecasis GR, Cherny SS, Cookson WO, Cardon LR (2002). Merlin--rapid analysis of dense genetic maps using sparse gene flow trees. Nat Genet.

[B16] Lander E, Kruglyak L (1995). Genetic dissection of complex traits: guidelines for interpreting and reporting linkage results. Nat Genet.

[B17] Cannella M, Gellera C, Maglione V, Giallonardo P, Cislaghi G, Muglia M, Quattrone A, Pierelli F, Di Donato S, Squitieri F (2004). The gender effect in juvenile Huntington disease patients of Italian origin. Am J Med Genet B Neuropsychiatr Genet.

[B18] Hubbard T, Andrews D, Caccamo M, Cameron G, Chen Y, Clamp M, Clarke L, Coates G, Cox T, Cunningham F, Curwen V, Cutts T, Down T, Durbin R, Fernandez-Suarez XM, Gilbert J, Hammond M, Herrero J, Hotz H, Howe K, Iyer V, Jekosch K, Kahari A, Kasprzyk A, Keefe D, Keenan S, Kokocinsci F, London D, Longden I, McVicker G, Melsopp C, Meidl P, Potter S, Proctor G, Rae M, Rios D, Schuster M, Searle S, Severin J, Slater G, Smedley D, Smith J, Spooner W, Stabenau A, Stalker J, Storey R, Trevanion S, Ureta-Vidal A, Vogel J, White S, Woodwark C, Birney E (2005). Ensembl 2005. Nucleic Acids Res.

[B19] Rangone H, Poizat G, Troncoso J, Ross CA, MacDonald ME, Saudou F, Humbert S (2004). The serum- and glucocorticoid-induced kinase SGK inhibits mutant huntingtin-induced toxicity by phosphorylating serine 421 of huntingtin. Eur J Neurosci.

[B20] Stephan D, Bon C, Holzwarth JA, Galvan M, Pruss RM (1996). Human metabotropic glutamate receptor 1: mRNA distribution, chromosome localization and functional expression of two splice variants. Neuropharmacology.

[B21] Maiese K, Vincent A, Lin SH, Shaw T (2000). Group I and group III metabotropic glutamate receptor subtypes provide enhanced neuroprotection. J Neurosci Res.

[B22] MacDonald ME, Vonsattel JP, Shrinidhi J, Couropmitree NN, Cupples LA, Bird ED, Gusella JF, Myers RH (1999). Evidence for the GluR6 gene associated with younger onset age of Huntington's disease. Neurology.

[B23] Rubinsztein DC, Leggo J, Chiano M, Dodge A, Norbury G, Rosser E, Craufurd D (1997). Genotypes at the GluR6 kainate receptor locus are associated with variation in the age of onset of Huntington disease. Proc Natl Acad Sci U S A.

[B24] Faber PW, Barnes GT, Srinidhi J, Chen J, Gusella JF, MacDonald ME (1998). Huntingtin interacts with a family of WW domain proteins. Hum Mol Genet.

[B25] Zhou R, Snyder PM (2005). Nedd4-2 phosphorylation induces serum and glucocorticoid-regulated kinase (SGK) ubiquitination and degradation. J Biol Chem.

[B26] Broman KW, Murray JC, Sheffield VC, White RL, Weber JL (1998). Comprehensive human genetic maps: individual and sex-specific variation in recombination. Am J Hum Genet.

